# Usability of a Mobile App for Improving Literacy in Children With Hearing Impairment: Focus Group Study

**DOI:** 10.2196/16310

**Published:** 2020-05-28

**Authors:** Shelly DeForte, Emre Sezgin, Janelle Huefner, Shana Lucius, John Luna, Anand A Satyapriya, Prashant Malhotra

**Affiliations:** 1 Research Information Solutions and Innovation The Abigail Wexner Research Institute Nationwide Children's Hospital Columbus, OH United States; 2 Clinical Therapies Department Nationwide Children's Hospital Columbus, OH United States; 3 OhioHealth Riverside Methodist Hospital Columbus, OH United States; 4 The Hearing Program in the Pediatric Otolaryngology Department Nationwide Children's Hospital Columbus, OH United States

**Keywords:** hearing aids, focus groups, cochlear implants, literacy, reading, hearing loss, hearing impairment, mobile applications, qualitative study, usability, aural rehabilitation

## Abstract

**Background:**

Children with hearing loss, even those identified early and who use hearing aids or cochlear implants, may face challenges in developing spoken language and literacy. This can lead to academic, behavioral, and social difficulties. There are apps for healthy children to improve their spoken language and literacy and apps that focus on sign language proficiency for children with hearing loss, but these apps are limited for children with hearing loss. Therefore, we have developed an app called Hear Me Read, which uses enhanced digital stories as therapy tools for speech, language, and literacy for children with hearing loss. The platform has therapist and parent/child modes that allow (1) the selection of high-quality, illustrated digital stories by a speech-language pathologist, parent, or child; (2) the modification of digital stories for a multitude of speech and language targets; and (3) the assignment of stories by a therapist to facilitate individualized speech and language goals. In addition, Hear Me Read makes the caregiver a core partner in engagement through functionality, whereby the caregiver can record video and audio of themselves to be played back by the child.

**Objective:**

This study aimed to evaluate the user experience of the Hear Me Read app through a focus group study with caregivers and their children.

**Methods:**

We recruited 16 participants (8 children with and without hearing loss and 8 caregivers) to participate in 1-hour focus groups. Caregivers and children interacted with the app and discussed their experiences through a semistructured group interview. We employed thematic analysis methods and analyzed the data. We used feedback from the focus group to improve the elements of the app for a larger clinical trial assessing the impact of the app on outcomes.

**Results:**

We identified three themes: default needs, specific needs, and family needs. Participants found the app to be esthetically pleasing and easy to use. The findings of this study helped us to identify usability attributes and to amend app functionalities to best fit user needs. Caregivers and children appreciated the enhancements, such as highlighting of parts of speech and caregiver reading of video playback, which were made possible by the digital format. Participants expressed that the app could be used to enhance family reading sessions and family interaction.

**Conclusions:**

The findings from this focus group study are promising for the use of educational apps designed specifically for those with hearing loss who are pursuing listening and spoken language as a communication outcome. Further investigation is needed with larger sample sizes to understand the clinical impact on relevant language and literacy outcomes in this population.

## Introduction

### Background

Nearly 2 to 3 per 1000 newborns are born with hearing loss, making hearing loss one of the most common birth anomalies [1]. Approximately 1 to 3 million children in the United States, and 34 million children worldwide suffer from disabling hearing loss [2]. Children with hearing loss face challenges in developing spoken language and literacy. Historically, the reading skills of deaf children have been poor, with graduating teenager reading scores comparable with first- to fourth-grade reading level [3-5]. Infants with hearing loss can now be identified at birth and fit with hearing aids and cochlear implants early in life, allowing them to have greater access to sound and improved oral language abilities [6-9]. However, even with modern hearing technology, many children who are deaf or have hearing loss may continue to read at significantly lower levels than typically hearing peers [10-12]. Young children who do not attain early literacy skills are at a higher risk for academic and social problems [13-16].

To address these challenges, we have developed a mobile app called the *Hear Me Read*. The National Association of Education of Young Children and other educational institutions emphasize the importance of reading storybooks to young children to enhance literacy [17-20]. In line with this recommendation, our intention with Hear Me Read is to use digital stories as therapy tools for speech, language, and literacy and to develop a platform for delivery that enhances family engagement for children with hearing loss. The Hear Me Read app is not meant to replace traditional storybook reading, but rather to enhance the user experience by providing the additional content individuals with hearing loss are expected to benefit from (eg, lip reading and audio-visual combination).

### Current Practice and Technology Use in the Education of Children With Hearing Impairment

Mobile phone and tablet technologies with digital electronic books (eBooks) and storybook reading are now commonplace in modern homes and schools. The impact of this technology on emerging literacy can be positive. However, digital eBooks can also be distracting when compared with traditional print storybook reading [21]. The impact of digital eBooks on the shared-book reading experience could be harmful if it increases distractions and reduces face-to-face interaction [22]. Thus, the design of *educational* apps should be done thoughtfully [23], especially in vulnerable populations such as children with hearing loss, with a focus on minimizing distractions and design flaws to promote engagement.

Although many apps deal specifically with certain parts of speech such as articulation, phonics, vocabulary, grammar, and comprehension, only a few are designed for children with hearing loss (Multimedia Appendix 1 shows a list of these apps). Furthermore, although sign language apps exist for children who are deaf or have hearing loss, there are no apps that are designed to develop spoken language and literacy in children with hearing loss pursuing a listening and spoken language outcome. The current *gold standard* for children with hearing loss to develop spoken language and literacy is through in-person one-on-one therapy sessions with a pediatric hearing loss expert (speech and language therapist or auditory-verbal therapist) [24]. Hear Me Read is developed to work specifically in the direction of a speech-language therapist, using digital stories as therapy extenders. Our app fits the principle of auditory-verbal intervention, where the caregivers are coached to be the *primary language facilitators* of their child’s language and literacy skills. When coaching is carried over into the home setting, we observe the most significant progress. Our app is an extension of this philosophy by enabling caregivers and their children to complete therapy activities outside of therapy sessions.

Children with hearing loss vary widely in their auditory access and how they acquire language or literacy skills [25]. They may benefit from multiple presentation modalities, including auditory, visual, or a combination of these approaches [26]. However, data regarding the efficacy of digital storybook interventions targeting children with hearing loss or how these children use existing digital reading technology are scarce. In this study, our objective was to understand user needs and expectations and the usability of Hear Me Read. The findings from this study were used to inform the app’s design process and improve the interface and functionality. Our next step will be to test Hear Me Read in a prospective study of children with hearing loss by measuring the impact of app usage on language and literacy outcomes.

## Methods

### Hear Me Read App

Hear Me Read is an interactive mobile app for improving language and literacy, which is targeted for auditory-based learning for children with hearing loss. Hear Me Read is composed of general features for improving literacy, including interactive storybook reading and syntax highlighting, audio-visual features including video recording and playback functionality, and a therapist mode, for the creation of individual therapeutic language and literacy goals. Some of the features, such as the ability to highlight and interact with vocabulary words that show a related image in Hear Me Read, are broadly applicable to children with and without hearing loss, whereas others, such as the playback of caregivers’ recorded video narration or the ability to highlight particular auditory training words, are specific to children with hearing loss.

**Figure 1:**
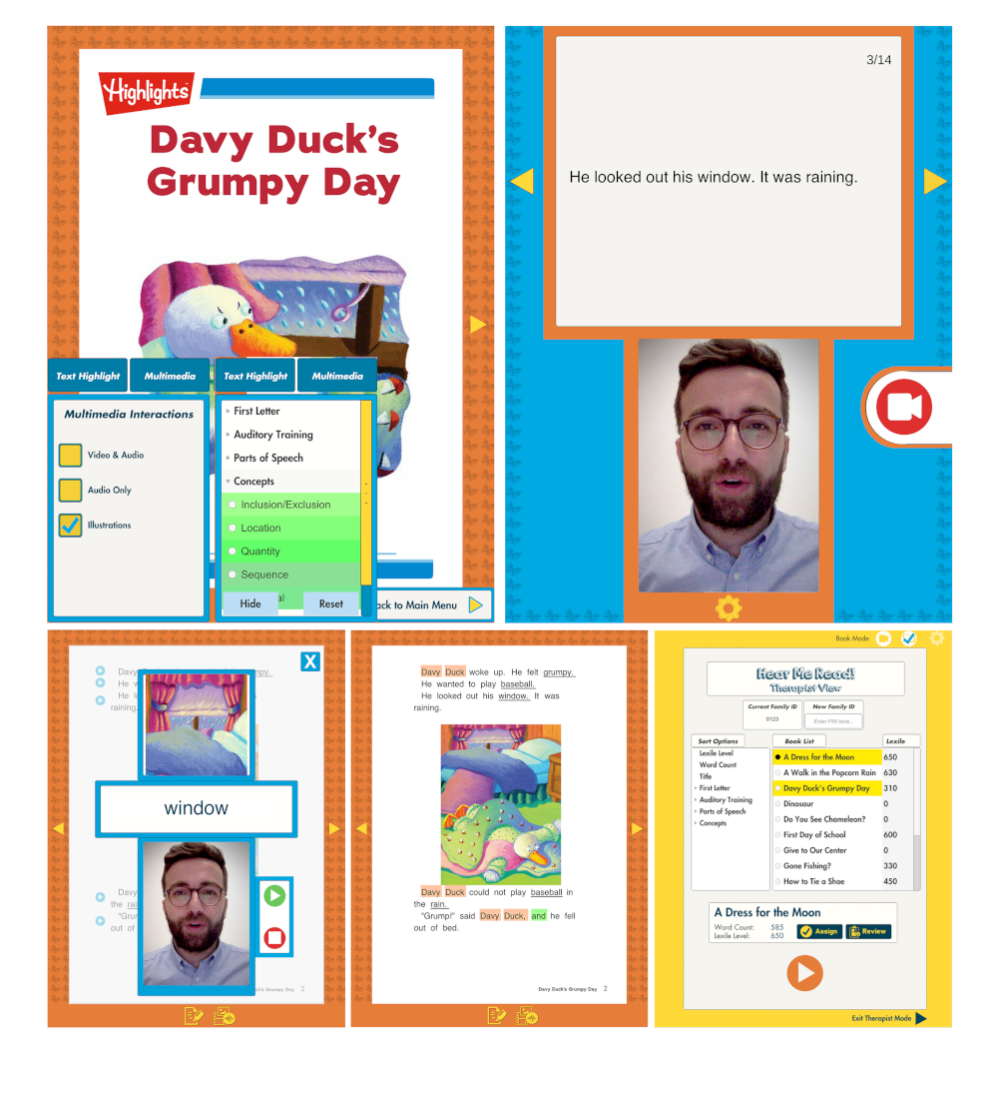
Screenshots from the Me Read app. The app provides multiple user menu options based on the therapeutic objectives (upper left). The app allows caregivers to record video and audio of themselves reading sentences and words (upper right), and caregivers and children can play back this video and view other associated media (lower right) during reading sessions. The app gives users the option to highlight parts of speech for targeted learning (lower middle) and a view for therapists to select custom learning objectives (lower right).

Hear Me Read provides an interactive digital environment for caregivers and children, using high-quality children’s stories (provided by Highlights for Children, Inc) in multiple formats and modalities (Figure 1). Hear Me Read allows the same digital story to be read in multiple ways: (1) text alone, (2) with illustrations, (3) with highlighted text targets, and (4) with audio and/or video recording of the parent reading (customized to display the text for the narrator to read and place it in a position relative to the camera that would produce a video where the narrator seems to be looking directly at the user).

Furthermore, Hear Me Read can help caregivers and therapists track reading progress and prescribe new reading assignments. In-app metrics can also measure the time spent in the book, the number of times read, and progress within the book. With these fundamental features, Hear Me Read is a one-of-a-kind app for children with hearing loss that leverages child-caregiver engagement. The development of the app used an iterative and user-centered approach. The layout of the user interface went through a few iterations during development and internal testing, as we narrowed down the scope of the project. Through observation and anecdotal feedback, we positioned and reshaped buttons to match the natural hand positions and interaction instincts of the users. A video introduction for the app is available in Multimedia Appendix 2.

The app was developed for the iPhone operating system using Unity. The app is intended to be used with the built-in speakers of the mobile device it is played on, and no additional calibration is provided for use with headphones. The digital content for the children’s book uses stories created by and retrieved with permission from Highlights Inc. We made a parser to translate the HTML exports of the PDFs of the Highlights books to a format that we could push into Unity. All content interactions are logged in the background and can be exported in comma-separated values (.csv) document format. The log files include user interaction event type, time of occurrence, and relative event state (eg, true/false, number, string). The event types include book open/close, book completion, narration start/stop, word click, app launch/close, video enable/disable, images enable/disable, audio enable/disable, highlight enable/disable, book assigned/unassigned, and Ling 6 sound [27]. Hear Me Read is currently not available for consumer use; however, this app will undergo further study in children with hearing loss to assess how it impacts language and literacy outcomes.

### Focus Group Study Design

After approval of the Nationwide Children’s Hospital’s (NCH) institutional review board, we recruited children with and without hearing loss and their caregivers. We included children without hearing loss because it allows us to explore usability across all literacy and hearing levels. Hearing loss can go from near-normal to completely deaf, and literacy capabilities can go from far below or far above peers. In clinical assessment, we would have to separate groups, but we wanted to make sure we had the entire spectrum of abilities and ages for the usability part. Following a demonstration and a short trial period of the Hear Me Read app, caregiver-child groups participated in a focus group discussion. Semistructured interviews were held among caregiver-child groups. This method is suitable for collecting rich information to understand the needs and expectations of families toward a technology-based solution to improve language and literacy skills for children with hearing loss. Observing caregiver-child relationships and engagement with the technology is also helpful. The session objectives were to identify obstacles that may interfere with the regular use of the app; to verify that participants can interface with and find value in the reading, recording, and language tasks; and to understand how the app may influence users and fit within the daily life of caregiver-child groups. The coauthors, SD and ES, designed and moderated the interviews. PM and JH helped recruit participants.

### Participants

Participants were recruited from a tertiary children’s hospital system. They were drawn from the Hearing and Cochlear Implant program or speech-language therapy department or were healthy volunteers. Participants—children and their caregivers—were invited via phone calls or during clinic visits. Participants were informed about the study aims, voluntary participation, and financial compensation. Gift cards were provided to families who participated in the study.

### Data Collection

A total of 2 focus group sessions were held in a pediatric therapy room for approximately 1 hour each. Before the focus group sessions, JH provided a written description of the study and collected consent forms from caregivers. Caregivers were then asked to complete a questionnaire (Multimedia Appendix 3) that provided information about child and caregiver hearing status, reading habits, and use of technology. The focus groups began with a brief introduction of the study and the team, study aims, participant’s rights, and the agenda. Participants watched a short video, which demonstrated the app (Multimedia Appendix 2). Then, an assigned speech therapist assisted every 2 to 3 caregiver-child groups to use and test the app together. Families were encouraged to use the Hear Me Read app without additional guidance, and speech therapists primarily observed and answered questions as needed. Participants were provided iPads with the Hear Me Read app installed, and participants engaged with the app using the iPad speakers for playback. For 20 min, caregiver-child groups explored the app and completed the tasks of (1) recording a video, (2) selecting highlights for words and letters, and (3) reading a book. Following app use, the study administrators facilitated a semistructured focus group interview. The focus group participants (both children and caregivers) were first asked usability questions concerning the app’s design and layout, functionality, ease of use, learnability, satisfaction, future use, and system reliability [28]. Usability questions were followed by questions intended to help understand the caregiver-child relationship with technology and use (eg, What technologies do you use at home? Which apps do you use mostly on your phone? Have you used any apps related to hearing before?). At the end of the session, caregiver-child groups filled out reaction cards together [29], where they highlighted the words from a list that express their feelings and opinions toward the app. The focus group sessions were audio recorded and transcribed.

### Analysis

We employed a thematic analysis to analyze the data collected during the study. Thematic analysis is a common approach in qualitative research to identify, assess, and analyze the patterns in the data [30] and is commonly used to evaluate usability for mobile apps [31,32]. The recorded audio of the focus group sessions was transcribed, and meeting notes and observational notes were curated into a single document. Coauthors ES and SD implemented inductive thematic coding on Microsoft Excel software (Microsoft, Inc) following Braun and Clarke’s [30] thematic analysis guideline. The following steps were used in the analysis process: (1) familiarizing with the data, (2) generating initial codes, (3) searching for themes, (4) reviewing and refining themes, (5) defining and naming themes, and (6) reporting the findings [30]. ES and SD went through multiple readings to extract codes and themes. Memos and observational notes were used to elaborate on the analysis. After independently developing themes and subthemes, ES and SD compared their independent coding schemes and agreed upon the 3 main themes used in the analysis and the division of individual comments. ES and SD then independently recoded 74 participant comments into 3 consensus themes (default needs: 26 items; specific needs: 34 items; and family need: 14 items). Cohen kappa inter-rater reliability testing was employed to ensure rater agreement for the themes (Multimedia Appendix 4) [33]. The scores were in the range of 0.81 to 1.00, which counted as an *almost perfect* agreement for each theme [33] with a highly significant *P* value (95% CI 0.57-1.0; *P*<.001), which means the agreement is significantly different from what would be achieved by chance. We used RStudio 1.2 as statistical software (RStudio, Inc). The authors employed 2 additional sessions to discuss and build a consensus upon themes and codes with the coauthors, where discrepancies were present.

## Results

### Demographics

In total, 8 caregiver-child groups participated in the study (caregiver: n=8; children: n=8) divided into 2 focus groups (group 1 and group 2), with the younger children (aged 2-5 years) in group 1 and the older children (aged 7-13 years) in group 2. One child attended a session with 2 caregivers, and another single caregiver attended a session with 2 children. Demographic and hearing loss information for the child participants were collected (Table 1). The children’s average age was 7.3 years (SD 3.5; range 2.4-12.4 years; median 7.2). Four (4/8, 50%) children were female. Five children (5/8, 63%) had moderate-to-severe sensorineural hearing loss, and all these children managed their hearing loss with bilateral cochlear implants. The caregivers of the children all used spoken language as the primary mode of communication at home, and 13% (2/15) of caregivers at home (1 child had a single caregiver at home) had hearing loss.

**Table 1:** Participant demographics.

ID	Group	Age (years)	Gender	Age ID^a^ (months)	Hearing age^b^ (months)	Management		Sensorineural hearing loss severity			CG^c^ HL^d^		
						R^e^	L^f^		R	L		1	2
1	1	2.4	F^g^	4	25	CI^h^	CI		Sev-Prof^i^	Sev-Prof		No	No
2	1	4.8	F	36	36	CI	CI		Mod-Sev^j^	Mod-Sev		No	No
5	1	3.7	M^k^	0	4	CI	CI		Profound^l^	Profound		No	No
3	2	11.3	F	0	6	CI	CI		Profound	Profound		No	No
4	2	9.3	M	0	4	CI	CI		Profound	Profound		Yes	No
6	2	12.4	F	N/A^m^	N/A	None	None		NH^n^	NH		No	No
7	2	7.3	M	N/A	N/A	None	None		NH	NH		No	No
8	2	7.0	M	N/A	N/A	None	None		NH	NH		Yes	N/A

^a^Age ID: age of diagnosis of hearing loss.

^b^Hearing age: age of cochlear implantation.

^c^CG: caregiver.

^d^HL: hearing loss.

^e^R: right.

^f^L: left.

^g^F: female.

^h^CI: cochlear implant.

^i^Sev-Prof: severe to profound hearing loss (>70 dB to 91 dB HL).

^j^Mod-Sev: moderate-to-severe hearing loss (>40 dB to 70 dB HL).

^k^M: male.

^l^Profound: Profound hearing loss (>91 dB HL).

^m^N/A: not applicable.

^n^NH: normal hearing.

### Technology Use

Questionnaire information regarding digital technology use in children in this study is mainly descriptive, given the small numbers (Table 2). All but one family used digital devices at home, although these devices were usually not used for digital reading.

Participants were given a survey before they interacted with Hear Me Read that asked about their current experiences with apps for hearing loss and features that they would ideally like in a reading app (Table 3). Participants are using training apps that provide visual and audio educational components. All caregivers of children with hearing loss knew what Ling 6 sounds were, and no children with normal hearing did (Table 3).

**Table 2:** Digital technology use.

ID	Device use at home		Apps commonly used			Digital reading device		
	Devices	Primary use at home	Caregiver app use	Child app use	Own?		Device	Reading done digitally
1	N/A^a^	N/A	N/A	N/A	Yes		Smartphone	None
2	Television^b^, smartphone	Videos	Social media, news	ABC Mouse, YouTube Kids	No		N/A	N/A
3	Television smartphone^b^	Work	Text (Telegram)	YouTube Kids	No		N/A	N/A
4	Television, computer, iPad, smartphone^b^	Web, communication, music	Navigation (Mapquest, Waze)	Music, games, videos	No		N/A	N/A
5	iPad^b^, smartphone^b^	Apps for speech, Netflix, work	Social media	Games	Yes		Kindle	1 in every 20 reading session
6	Television, computer, Kindle, smartphone^b^	News, communication, social media, music, videos	Web, email, text, Facebook, running tracking, music	Games, camera, Kindle	Yes		Kindle	6 in every 10 reading sessions
7	Television, computer, Kindle^b^, smartphone	News, communication, social media, music, videos	Web, email, text, Facebook, running tracking, music	Games, Kindle	Yes		Kindle	2 in every 10 reading session
8	Television, iPad^b^	Learning	Facebook	iConnection	No		N/A	N/A

^a^N/A: not applicable.

^b^Devices most frequently used.

**Table 3:** Hearing loss app use.

ID	Apps used for hearing loss	Read differently because of hearing loss? In what way?	What would you like to have in a reading app?	Know Ling 6 sounds?
1	None	Yes—“We have done signs quite a bit with reading; Using our fingers to point out each word as we read.”	“Show the sign after I reads it. That way, when a child knows the sign it can make the link from the sign to the audio part of the object; repeat on the page.”	Yes
2	None	Yes—“I break down every part to make sure she understands what is happening.”	“It would need to grab their attention. Also make them feel like they could understand and work the app.”	Yes
3	My Signing Time, Sign and Sing	No	“An app that needs a passcode to exit the app while using it. Videos that demonstrate the task that is being teached. Vivid colors.”	Yes
4	Lexia, RazKids	N/A^a^	“At this age, I would say to help (name) pronounce more challenging words and provide definitions of their meaning as she is reading.”	Yes
5	Speech Stickers, Hope Words, Kids Vocab-Read Comp 1	No	“Have it have precise speech comprehension questions at the end.”	Yes
6	None	N/A	N/A	N/A
7	None	N/A	N/A	N/A
8	None	N/A	“To slowly pronounce words as they are said and seen on screen.”	N/A

^a^N/A: not applicable.

### Focus Group Discussion Themes

Focus group analysis identified 3 major themes. The first theme, *default needs*, represents the generic needs from an app. The subthemes are ease of use, the intuitiveness of navigation, layout, and workflow, with comparisons to generic apps. The second theme, *specific needs*, represents the needs regarding hearing loss. The subthemes are the reading and language comprehension functionality, user engagement, and preferences, including attention, learning, and design. The third theme, *family needs*, includes family suggestions about how the app could be used, family interactions, and how Hear Me Read might fit within the daily lives of the participants. The subthemes are family relationships and daily life.

#### Theme 1: Default Needs (Ease of Use Compared With Generic Apps and Identifying Common Needs)

##### Ease of Use

Participants generally found the app to be easy to use with a pleasing design. Most participants agreed that the app compared favorably with other apps that they used frequently. Despite the difficulty in use because of the new components and complexity of functions, users confirmed that Hear Me Read is easy to learn and a fulfilling app at the first use. Caregivers and children were able to overcome difficulties and navigate the app after watching the short introductory video and briefly exploring the app:

..., did you find doing the tasks difficult? Was it easy and intuitive to learn it? Was there anything you got stuck on?

facilitator, group 1

No, just after I clicked around a little.

caregiver, group 1

Yeah, once you’re exploring, you kind of get the hang of it.

caregiver, group 1

##### Navigation

However, 2 functions are presented for modifying the usability of the app. The first was the page-turning functionality. Initially, the app required a long swiping motion across the bottom of the screen to navigate to a new page. However, participants, especially young children, found this method of navigation to be complicated:

Children I think are going to really struggle. I think with flipping pages, if you have to have your finger in the small little corner to get it over, instead of just hitting a button to go to the next page or anywhere on the screen.

caregiver, group 1

The chief complaint seemed to be that the swiping motion was too specific and not forgiving enough when general swiping movements or single taps were used. The children who were using the app tended to instinctively press a single point to navigate to the next page:

Yeah, at first I just tapped it, and I thought it was like, if you tap it, it’ll go.

child, group 2

However, caregivers mentioned that they tended to see children using a swiping motion more often for other apps:

I think kids in general, when they think of any kind of device they just kinda go like this [slides a finger from bottom to top of iPad] and it’s just what they’re used to doing.

caregiver, group 2

##### Layout and Workflow

The second function with noted concerns was the recording function, which required the user to click record on every page. This action was not intuitive to most caregivers and caused them not to record once they progressed past the first page:

I didn’t realize you had to hit the record button after every page. We just kept right on going.

caregiver, group 2

The app allows the ability to playback either video or audio. Although some participants only engaged with the audio, others did not realize that audio only was an option. In addition, some participants did not comprehend that the recording could be deleted and replaced.

#### Theme 2: Specific Needs (Core Functionality, Engagement, and User Preferences)

##### Reading and Language Functionality

Participants mentioned multiple times the ability to highlight parts of speech as a favorite feature:

I liked the feature of highlighting the word, vocabulary, stuff like that. That was a good idea.

caregiver, group 1

I loved how you could highlight pronouns and verbs and adjectives, and I see where that could be helpful with both of my children, both hearing and non-hearing. I think that’s a real plus of the app.

caregiver, group 2

##### User Engagement

However, groups 1 and 2 engaged in the game differently. For younger children (group 1), the video of a caregiver was especially engaging:

When she did see me talking though, she did get really excited at first, like “I see mommy.”

caregiver, group 1

In addition, caregivers of younger children expressed concern that the text was too dense, and visual interest was insufficient to maintain engagement. Participants suggested increasing visual interest for younger children through pictures, colors, and font choices:

I think if the book was longer and had some pictures... for each sentence have like a picture of what...the story is trying to tell. Cause he [referring to son] got excited when he saw the photos.

caregiver, group 1

I feel like since it’s for a kid, it should have more color to attract kids.

caregiver, group 1

He’s still reading books that are kind of fun to look at. Even chapter books that he reads, the font is a more playful font, and it’s just more, I think, eye-catching for them.

caregiver, group 2

##### User Preferences

Older caregiver-child groups wanted more advanced features, chapter books, and quizzes for comprehension:

I think maybe you should add like a little quiz at the end or like something, just kinda refresh your memory.

child, group 2

.*..so to have a couple questions at the end...that would make him think about what he just read*. [caregiver, group 2]

Although the app is intended for caregivers to record themselves reading, some caregivers also suggested that the children could record themselves:

Or you could even work with them, and they could even learn a sentence and record it themselves doing it; so I thought that was cool, too. Once they learn the word, you could have them do the book and then play it back to them.

caregiver, group 1

However, a caregiver also noticed that it was very distracting for her child to record himself:

I like the fact that I can record myself, but that it’s extremely distracting for him to record himself. All he wants to do is look at himself...very distracting for him.

caregiver, group 2

#### Theme 3: Family Needs (Integration of the App in Family Relationships and Daily Life)

##### Family Relationships

Participants emphasized the value of family engagement and joint use of the app for education and training of the children. In that regard, they agreed that they liked the use of custom videos of family. Participants also suggested that they could use the app to include extended family members (eg, grandmother) for video recording, which can help to bond family members through this media:

And that my face is on there, that he recognized. It’s not like some random voice or a stranger’s face or something, I like that.

caregiver, group 2

But to your point, at least it could be a family member this way.

caregiver, group 2

Yeah! Like a grandparent, that is a way they could help them.

caregiver, group 2

So, you recognize the voice, it’s not like some random person.

child, group 2

##### Daily Life

Caregivers cited after school and bedtime as the most likely time for interaction with the app and estimated that they would spend 15 to 30 min interacting with their child and the app each day:

Well he started reading in school, so we would probably use it for after homework or something.

caregiver, group 2

If you had a parent who traveled, I think it would be wonderful to re-record bedtime stories, and they see you. I mean, that is a really nice thing!

caregiver, group 2

Some caregivers referenced current reading times for their children and suggested that they could use the app during these times:

He has required nightly reading so that would be nice.

caregiver, group 2

I feel like before bedtime would be easiest when he does his reading.

caregiver, group 1

### User Reactions

**Figure 2:**
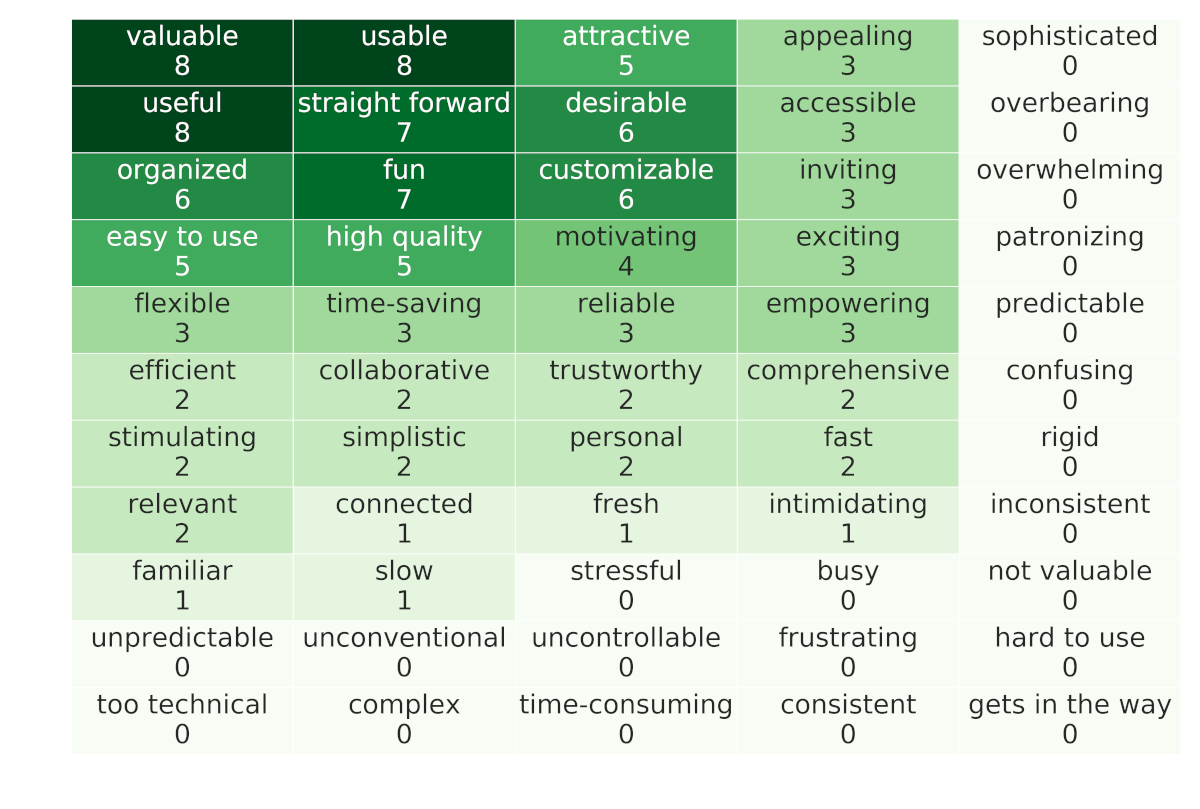
User reactions. Users were asked to mark words that they associated with the app. Displayed are the total counts for each word that was marked. Words were rearranged by count for display.

At the end of the interview session, participants were given reaction cards with a series of words and asked to mark each word that they felt applied to the app. This method was used to quickly capture their thoughts and feelings about the app. In Figure 2, the reactions are arranged from the highest number of marks in the upper left corner to the lowest at the bottom right of the figure. The highest number of marks was found for those words most related to ease of use (usable, easy to use, and straight forward) and value (valuable, useful, and motivating). In addition, words related to positive design aspects had high and medium representation (desirable, attractive, appealing, and inviting). The words *intimidating* and *slow* each had one mark, whereas other negative words received no marks.

## Discussion

### Overview

Hear Me Read is an app built to expand upon the *gold standard* practice for developing literacy in children with hearing loss, which consists primarily of in-person therapy sessions with a pediatric hearing loss expert. Hear Me Read allows caregivers to work with therapists to develop lesson plans within the app, thereby facilitating at-home interactions between caregivers and their children regularly. Furthermore, the recording functionality allows the caregiver to prerecord reading sessions that the child can use alone. Children can, therefore, interact with the text in multiple ways. Caregiver and child engagement while using this technology is the core objective of this app. Joint caregiver-child engagement with media facilitates learning [34] and can enhance family relationships [35]. Few commercially available apps promote the caregiver’s involvement while the child is engaging with the app. Hear Me Read is developed with a user-centered perspective and makes the caregiver a core partner in engagement through functionality, whereby the caregiver can record video and audio of themselves to be played back by the child, assign customized reading objectives, and read together with their child. Hear Me Read’s utility is designed to supplement the joint book reading in which caregivers are already engaging their children. Hear Me Read is not designed to replace traditional printed storybooks. Caregivers can apply the knowledge from the Hear Me Read app to their shared storybook reading experiences using a variety of platforms, including both printed text and digital text.

The objective of this study was to use focus groups to investigate the usability of the Hear Me Read app within caregiver-child groups. Through participant interaction with the app followed by a focus group discussion, we sought to identify areas requiring modifications and improvements during the use of the app, to understand how caregivers and children interacted with the core functionality of the app, and to determine how they might use the app in their daily life.

### Principal Findings

We conducted two focus groups, one with children aged 2 to 5 years and their caregivers and the other with children aged 7 to 12 years and their caregivers. All children with hearing loss had bilateral cochlear implants. The majority of family groups used either smartphones or Kindle as their primary digital devices, although not all participants used these devices for digital reading. When considering the generic attributes of Hear Me Read, participants agreed that the app was visually pleasing, generally easy to use, and compared favorably to other apps they use (both general apps and apps related to literacy). We identified two obstacles (default needs) for ease of use in the app, namely the page-turning functionality and the recording functionality. Without a clear consensus on the preferred way to turn the page (swiping vs pressing), we changed the navigation to accept either a general swiping motion or a button press on the right or left side of the display. In addition, our participants tended to assume that recording continued as they changed the pages, so we also updated our app to allow for continuous recording rather than requiring it to be halted and then reinitiated at each page. Despite these two usability issues, participants marked the app high for ease of use in the reaction cards. The aforementioned favorable attributes and obstacles may be common issues for any user group. We have not identified any significant correlation with those who have hearing loss and these user preferences.

In terms of user-specific needs, a key objective was to determine if functionality deviates from non-digital standard practices. In that regard, participants found that customized options such as recording and playback and dynamic word highlighting were accessible and valuable. The participants also expressed appreciation for both the video recording and highlighting capabilities, suggesting that these features would be useful for improving literacy. Such user-level customization is a significant driving factor in the digital health app literature as well [36]. Most caregiver participants already included daily reading activities with their children, either after school or before bed, and were able to see using the app as fitting within their daily schedules for 15 to 30 min. Most of the participants expressed appreciation for the recording functionality and the ability to record a family member rather than to have a video of a stranger. They suggested that this feature may increase engagement because of familiarity and may also increase family bonding, even when caregivers and children are not reading together. This finding supports the importance of including coviewing design elements in development through joint media engagement with family members. This aspect will be investigated further in a clinical trial.

### Limitations

Participants in this study interacted with the app for only a limited time (40 min), which may have limited their experiences to be able to interact with all the features of the app. We observed that different participants interacted with different features of the app. However, none of the participants were able to interface with the full suite of features. Testing time constraints may have limited further investigation of usability issues. The two focus groups represented a relatively small sample size (n=8) and may not have been representative of the full spectrum of the patient and caregiver population. Furthermore, heterogeneity in terms of the children’s age and gender may limit the generalization of age- or gender-specific implications. The presence of children both with and without hearing loss means that our feedback was skewed toward more general features that are accessible to children with and without hearing loss. However, we found that the feedback we received from both groups was congruent.

The NCH clinic recruited participants with a snowball approach, and therefore, familiarity with some of the staff, including the speech therapists who were present during the focus group, may have had an inhibitory effect on negative feedback. Finally, the scope of this study was limited to an evaluation of the usability of core functions. We did not ask participants to consider evaluating all features (eg, the therapist tools) of the app in depth. Future studies should expand to evaluate all features with an extended user time, and therapeutic tasks should be assigned and personalized for each participant.

### Conclusions

We conducted a focus group study for usability testing of the Hear Me Read app. Participants primarily found the app to be easy to use, esthetically pleasing, and valuable. Feedback from this study was used to improve the app and contribute to the literature by reporting user needs and expectations from children with hearing loss and caregiver population for a mobile app. The findings are promising for the use of educational apps designed specifically for the hearing loss population. Further investigation is needed with larger sample sizes and the actual impact on relevant language and literacy outcomes in this population.

## Appendix

Multimedia Appendix 1 A table compiling existing apps for help with speech-language pathology, literacy, and hearing loss. Apps were found through a search on the Apple iTunes app store and a Google keyword search. The keywords for the search were related to speech therapy apps for children with hearing loss along with related terms, such as phonics, phonetics, and auditory memory.

Multimedia Appendix 2 A video explaining how to use the Hear Me Read app.

Multimedia Appendix 3 Questionnaire for parent participants in the focus groups.

Multimedia Appendix 4 Cohen kappa inter-rater reliability testing results.

## Abbreviations

eBook: electronic book
NCH: Nationwide Children’s Hospital
