# Supplementary material for: Usability of a Mobile App for Improving Literacy in Children With Hearing Impairment: Focus Group Study
Source: JMIR Hum Factors. 2020 May 28;7(2):e16310. doi: 10.2196/16310 (PMC7290449; doi:10.2196/16310)
Supplement: Multimedia Appendix 3 [file humanfactors_v7i2e16310_app3.docx]

**Hear Me Read! Prototype**

**Questionnaire – Parent**

**Child Name:** ____________________________ **Date:** ______________

**Child’s DOB: _______** **Gender:** M F *(circle)* **Visually impaired?** Yes No *(circle)*

**Age at ID of hearing loss:** _____ ___ **Age when hearing aid first put on:**_________________

**Child’s RIGHT Ear:**

Management: *(circle)* Cochlear Implant Hearing Aid Bone Anchored Device None

Type of Hearing Loss: Conductive Sensorineural Mixed Don’t Know Normal

Degree of Hearing Loss: Mild Moderate Severe Profound

**Child’s LEFT Ear:**

Management: *(circle)* Cochlear Implant Hearing Aid Bone Anchored Device None

Type of Hearing Loss: Conductive Sensorineural Mixed Don’t Know Normal

Degree of Hearing Loss: Mild Moderate Severe Profound

**Parent’s Name (completing the form)** _____________________________________________________

**Does Primary Caregiver have hearing loss?** Yes No *(circle)*

Primary Caregiver way of communicating: Spoken language ASL Both Other (specify): _________________

**Does Caregiver/Partner have hearing loss?** Yes No *(circle)*

Caregiver way of communicating: Spoken language ASL Both Other (specify): _________________

What technologies to you use at home (TV/computer/smartphone, etc.)?

Which one is used most frequently?

What is the technology used for mostly?

Which apps do you use mostly on your phone?

Which apps does your child use mostly on your phone?

Do you own a digital reading device? Yes No *(circle)* If so, what device? _______________

Approximately what percentage of reading is done on this device by your child? _________________

How often does **caregiver** access a computer for education?

0 1-4 5-7 (days/week); Average number of minutes/week _____

How often does **caregiver** access a computer for entertainment?

0 1-4 5-7 (days/week); Average number of minutes/week _____

How often does **caregiver** access a mobile device/tablet for education?

0 1-4 5-7 (days/week); Average number of minutes/week _____

How often does **caregiver** access a mobile device/tablet for entertainment?

0 1-4 5-7 (days/week); Average number of minutes/week _____

How often does **child** access a computer for education?

0 1-4 5-7 (days/week); Average number of minutes/week _____

How often does **child** access a computer for entertainment?

0 1-4 5-7 (days/week); Average number of minutes/week _____

How often does **child** access a mobile device/tablet for education?

0 1-4 5-7 (days/week); Average number of minutes/week _____

How often does **child** access a mobile device/tablet for entertainment?

0 1-4 5-7 (days/week); Average number of minutes/week _____

Child’s Lexile Level of Reading: _________; Grade Level _________ *(if known)* **or** don’t know

How often does your child read books for fun: 0 1-4 5-7 (days/week); Avg min/week ____

How often do caregivers read aloud with your child: 0 1-4 5-7 (days/week); Avg min/week ____

How many days a week do ***you*** read books for fun: 0 1-4 5-7 (days/week); Avg min/week ____

**Do you know what Ling sounds are?** Yes No *(circle)*

**Have you used any apps related to hearing loss before?** Yes No *(circle)*

If yes, which apps have been used?

If not, why have they not been used?

**Do you read differently to you child because of hearing loss?** Yes No *(circle)*

If Yes, in what way?

**What would you like an app to do that can help your child read or learn words?**
