# Supplementary material for: Usability of a Mobile App for Improving Literacy in Children With Hearing Impairment: Focus Group Study
Source: JMIR Hum Factors. 2020 May 28;7(2):e16310. doi: 10.2196/16310 (PMC7290449; doi:10.2196/16310)
Supplement: Multimedia Appendix 4 [file humanfactors_v7i2e16310_app4.docx]

Table. Themes, sub-themes and inter-rater reliability scores

| **Themes** | **Total items** | **Sub-themes** | **Inter-rater reliability score (Cohen’s Kappa)** | **p-value** | **95% CI lower bound** | **95% CI upper bound** |
| --- | --- | --- | --- | --- | --- | --- |
| Default needs | 26 | - Ease of use - Navigation - Layout - Workflow | 0.885 | .000** | 0.666 | 1.000 |
| Specific Needs | 34 | - Reading and language comprehension functionality - User engagement - Preferences | 0.941 | .000** | 0.827 | 1.000 |
| Family Needs | 14 | - Family relationships - Daily life | 0.851 | .001** | 0.573 | 1.000 |

** p < 0.001, CI: Confidence Interval
